# Papillary thyroid carcinoma with brain and lung metastases: a case report

**DOI:** 10.3389/fonc.2026.1849526

**Published:** 2026-05-25

**Authors:** Ying Zhao, Xiaohua Chen, Xin Bai, Shujun Guo, Min Gao, Xiaohan Liu, Jianming Tang

**Affiliations:** 1 The First Clinical Medical College, Lanzhou University, Lanzhou, Gansu, China; 2 Department of Radiation Oncology, The First Hospital of Lanzhou University, Lanzhou University, Lanzhou, Gansu, China

**Keywords:** anlotinib, brain metastases, lung metastases, papillary thyroid carcinoma, radioiodine-refractory differentiated thyroid cancer

## Abstract

**Background:**

Papillary thyroid carcinoma (PTC) usually has a favorable prognosis, the concurrent occurrence of brain and lung metastases is extremely rare, thus such cases have significant clinical reporting value. We report a case of a patient with multifocal PTC who presented with delayed multiple metastases.

**Case presentation:**

In January 2025, a 61-year-old female suddenly developed speech impairment and right limb weakness without an obvious cause. Upon reviewing her medical history, it was found that she had undergone a total thyroidectomy six years earlier because of multiple papillary thyroid carcinomas. Upon admission, relevant examinations were performed: cranial MRI revealed a solitary mass in the left frontal lobe, and a chest CT revealed multiple nodules in both lungs. The postoperative pathological examination of the brain lesion and bronchoscopic biopsy of the mediastinal lymph nodes revealed metastatic thyroid cancer (Pax-8, Tg, and TTF-1 positive). Diagnostic ¹³¹I whole-body scan after high-dose radioactive iodine treatment (180 mCi) revealed no significant radioactive isotope uptake by the lung metastases, indicating a radioactive iodine-resistant disease. Subsequently, three cycles of systemic chemotherapy (albumin-bound paclitaxel combined with carboplatin) were tried, but they were discontinued because of extreme toxicity.

**Treatment and results:**

The patient underwent surgical resection of brain metastases, and then switched to the multi-kinase inhibitor anlotinib due to poor chemotherapy tolerance. After approximately 10 weeks of anlotinib treatment, the first CT assessment showed a partial response, with the lung metastases shrinking and partially resolving. The most recent follow-up CT (in March 2026) confirmed that the remission was ongoing.

**Conclusion:**

This case indicates that PTC can result in uncommon, delayed, and numerous distant metastases even years after radical surgery. This highlights the importance of life-long surveillance, including chest and crinial imagings. The partial remission with anlotinib underscores its value as a therapeutic option in this setting.

## Introduction

Thyroid carcinoma is the most common endocrine malignant tumor (1). The metastatic behavior of thyroid cancer varies depending on the histological type, but distant metastasis is generally uncommon, especially in well-differentiated thyroid cancers such as papillary and follicular carcinomas (2). With a favorable prognosis, papillary thyroid carcinoma (PTC) is the most prevalent type of thyroid cancer, making up 84% of all cases. The incidence of local neck metastasis is relatively high in PTC, but only 1-4% of patients develop distant metastasis. Lung, bone, liver, and brain are the most common locations for distant metastases (3). Other unusual sites of metastasis include the occipital bone, internal jugular vein, parapharyngeal space, sternocleidomastoid muscle, and even the right atrium of the heart (4). This article examines a complicated case of a 61-year-old female who had metastases in her lungs and frontal lobe six years after having a thyroidectomy.

## Clinical history and pathology

**Figure 1** presents a detailed timeline of the patient’s clinical course. On January 7, 2025, a 61-year-old woman presented with a sudden onset of dysarthria without an apparent cause, with preserved comprehension. Despite having weakness in her right limb, she was able to walk normally. There were no concomitant symptoms of altered consciousness, slurred speech, or hearing loss. She sought care at another hospital, where subsequent a cranial MRI revealed a tumorous lesion in the left frontal lobe, suspected to be metastatic. The patient and her family came to our hospital for additional assessment and care.

**Figure 1:**
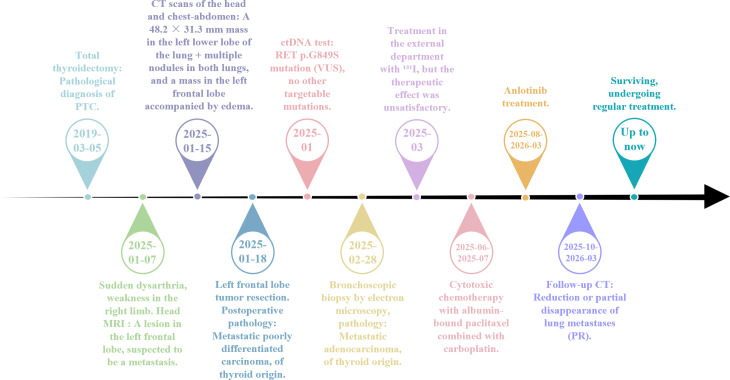
The timeline shows key events from the initial thyroidectomy in March 2019 to the last follow-up in March 2026.

The patient was admitted to the hospital with the following metrics: height of 164 cm, weight of 65 kg, body surface area of 1.68 m², NRS score of 0, and a KPS score of 80. The patient was well-nourished with normal gait, posture, and mental state. There were no abnormalities in composite sensation, and deep and superficial sensation are present in the limbs and trunk. Muscle tone and muscle tension are normal in the limbs. Physiological reflexes are present, and there are no bilateral pathological signs.

After multiple metastatic tumors were found, additional investigation into the patient’s history revealed that she underwent a total thyroidectomy at another medical on March 5, 2019. Postoperative pathology and immunohistochemical analysis confirmed papillary thyroid carcinoma. The surgical pathology report documented multifocal disease, including a dominant left cervical mass (45 × 30 × 30 mm) with a 10 mm nodule in the left lobe, an 8 mm papillary microcarcinoma in the right lobe. All dissected lymph nodes (central compartment, left levels 2, 3, and 4) were metastasis negative (0/9). Therefore, according to the 2025 American Thyroid Association (ATA) Management Guidelines for Adult Patients with Differentiated Thyroid Cancer, the patient’s pathological stage is pT3aN0M0, Stage II (5).

After the surgery, the patient was treated with regular oral levothyroxine sodium tablets (100 μg/day) and underwent routine follow-up thyroid hormone panel tests and imaging examinations, all of which showed no abnormalities. Prior to admission, the patient had no subjective symptoms. The patient had pulmonary nodules for two years, but no particular treatment had been administered. There is no history of hepatitis or tuberculosis, no infectious diseases, no known exposures, no vaccinations, no drug allergies, and no blood transfusions. In terms of personal history, the patient was married, had not resided in infectious areas, and had abstained from smoking, alcohol, and substance abuse, with no exposure to industrial toxins, dust, or radiation, and no travel history. No genetic diseases were reported in the family history.

A series of CT scans of the patient’s chest and abdomen on January 15, 2025, showed a soft-tissue density mass in the anterior-medial basal segment of the left lower lobe that measured about 48.2 × 31.3 mm. A neoplastic lesion was indicated by the presence of enlarged lymph nodes in the periphery. Multiple solid lesions of varying sizes were identified in both lungs, suggesting metastases (**Figure 2**). Additionally, a left frontal lobe mass with surrounding edema was discovered during a cranial CT scan on January 18, 2025 (**Figure 3**). A subsequent bone scan revealed no abnormalities. After the MDT discussion, the patient underwent a frontal lobe tumor resection on 18 January 2025. Postoperative pathology indicated: morphological and immunohistochemical findings consistent with metastatic poorly differentiated carcinoma, most likely of thyroid origin (**Figure 4**). The postoperative cranial CT scan confirmed that the left frontal lobe metastatic tumor had been completely removed and there were no residual enhanced tumors (**Figure 5**). As a result, it was determined to regularly conduct cranial MRI for monitoring, without performing auxiliary stereotactic radiosurgery (SRS) or whole brain radiotherapy (WBRT). The patient’s right-limb weakness significantly improved following surgery. Her dysarthria completely disappeared, and she was able to walk independently on the 5th day after the surgery, without any new neurological dysfunction occurring.

**Figure 2:**
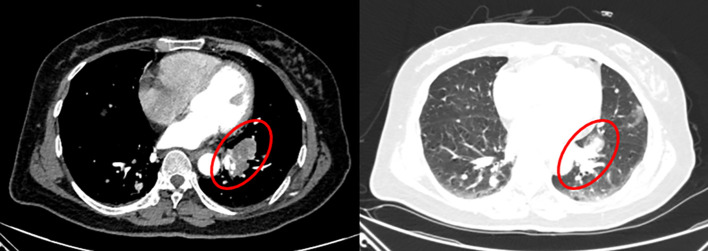
On 15 January 2025, a chest CT scan revealed the presence of a soft tissue density mass measuring approximately 48.2×31.3mm in the anterior-medial basal segment of the left lower lobe. Enlarged lymph nodes were observed in the periphery, indicative of a neoplastic lesion. Multiple solid lesions of varying sizes were identified in both lungs, suggesting metastases.

**Figure 3:**
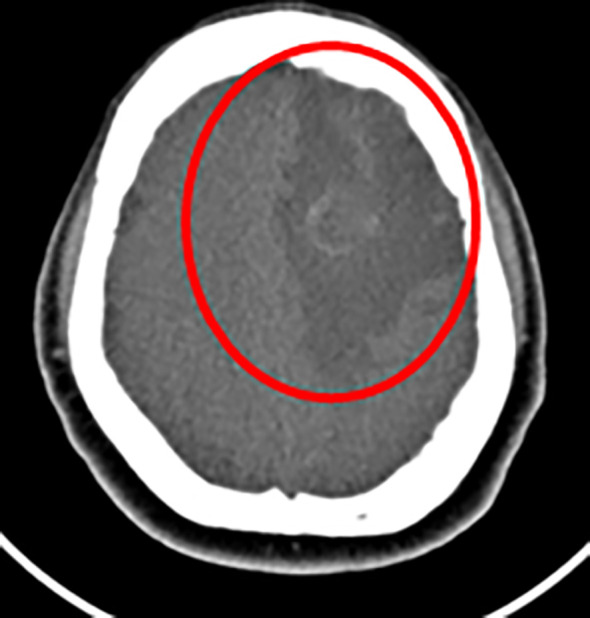
A cranial CT scan conducted on 18th January 2025 revealed a left frontal lobe mass accompanied by surrounding edema.

**Figure 4:**
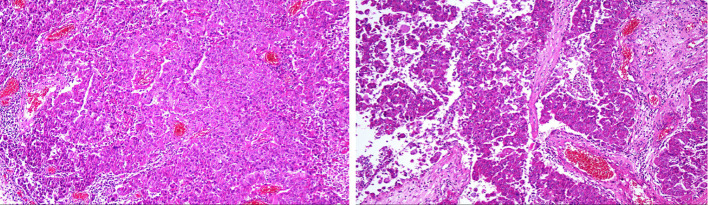
The post-op specimen measures 28 x 21 x 7 mm and shows gray-white to gray-red inelastic tissue. Diagnosed as a metastatic poorly differentiated carcinoma, most likely of thyroid origin.

**Figure 5:**
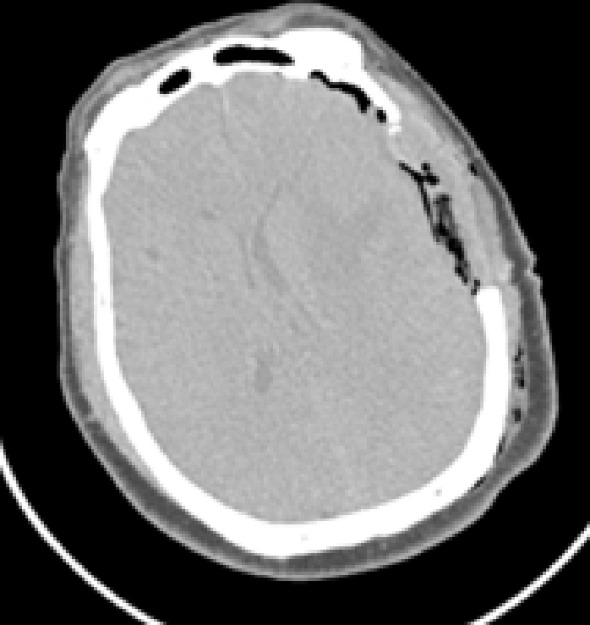
On January 18, 2025, the postoperative cranial CT scan of the left frontal lobe lesion showed that the left frontal lobe metastatic tumor had been completely removed, and there were no residual enhanced tumors.

To explore the molecular characteristics of the patient’s metastatic tumors and guide subsequent treatment, circulating tumor DNA (ctDNA) was analyzed. A RET p.G849S mutation with a variant allele frequency of 46.72% was identified by the genetic analysis. However, according to existing databases and literature, this variant is classified as of uncertain clinical significance (Category III). Moreover, there is currently no definitive evidence linking it to sensitivity to any RET-targeted therapy, rendering it clinically non-actionable for guiding targeted treatment at present. Additionally, no sensitivity or resistance mutations were detected in common driver genes such as EGFR, ALK, BRAF, or KRAS. Immunotherapy-related marker testing revealed no pathogenic mutations in mismatch repair (MMR) system genes (MLH1, MSH2, MSH6, PMS2), indicating a pMMR/MSS tumor phenotype. This finding indicates a predicted minimal benefit from monotherapy with immune checkpoint inhibitors. Pharmacogenomic analysis indicated that platinum-, taxane-, and pemetrexed-based regimens exhibited favorable efficacy, whereas gemcitabine and capecitabine demonstrated reduced efficacy. In summary, the present report confirms the absence of advantageous gene mutations suitable for targeted or immunotherapy, suggesting that conventional targeted agents and PD-1/PD-L1 inhibitors may be unsuitable for this patient (6, 7).

The patient went back to the hospital a month later for further care. An electronic bronchoscopy was conducted on February 28, 2025, and biopsy samples were then sent for pathological analysis. The results of lymph node stations 11Ri and 11L indicated that atypical epithelioid cells were observed, suggestive of metastatic adenocarcinoma. Immunohistochemistry indicated that the tissue of origin was most likely the thyroid (**Figure 6**). Therefore, this patient was finally diagnosed as having a malignant thyroid tumor, a secondary malignant tumor of the brain, and a secondary malignant tumor of the lung.

**Figure 6:**
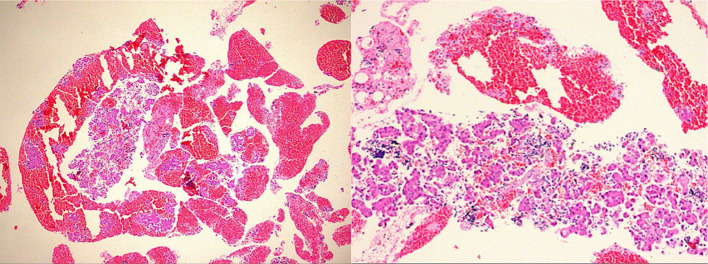
On February 28, 2025, the pathological and immunohistochemical results following electronic bronchoscopy showed atypical epithelial-like cells, suggesting metastatic adenocarcinoma. The most likely tissue origin was the thyroid gland.

Although it is well-established that PTC and its metastases largely retain sodium-iodide symporter (NIS) function, enabling radioactive iodine (¹³¹I) uptake. In addition, the 2022 ETA/2021 NCCN/2025 ATA guidelines stipulate that, in cases of PTC accompanied by distant metastases, ^131^I ablation therapy is recommended, contingent on the presence of iodine-avid lesions or the unresectability of the lesions. The patient was therefore recommended for ^131^I therapy.

The patient was treated with oral ^131^I-NaI at a dose of 180 mCi in March 2025 at another hospital. On the third day, whole-body scintigraphy and SPECT/CT fusion imaging were performed to evaluate iodine uptake. The imaging results showed no significant radioactive iodine uptake in bilateral pulmonary nodules or other metastatic sites (negative iodine affinity). Therefore, the treatment was considered ineffective, and the patient was diagnosed with radioiodine-refractory differentiated thyroid cancer (RAIR-DTC).

On June 5, 2025, a chest CT scan revealed that the pulmonary metastases were advancing following the RAIR-DTC diagnosis (**Figure 7**). Because the pharmacogenomics results showed that the platinum and taxane-based treatment regimens had good efficacy, the patient then received cytotoxic chemotherapy based on albumin-bound paclitaxel (260 mg/m²) combined with carboplatin (AUC 5). After three cycles of chemotherapy, severe chemotherapy-related toxic reactions occurred, including grade 3 neutropenia and severe fatigue, which greatly affected her physical condition and quality of life. During this period, a chest CT scan was re-conducted on July 21, 2025, indicating that the pulmonary nodules and masses showed no significant change compared with the prior scan (**Figure 8**).

**Figure 7:**
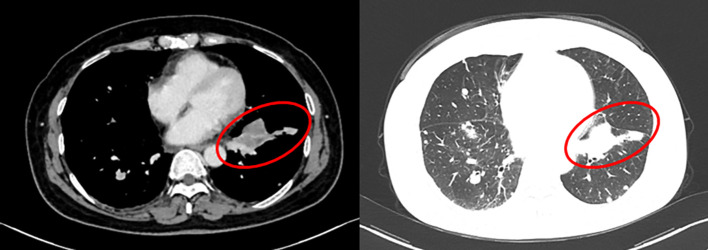
The chest CT scan conducted on June 5, 2025, revealed that the metastatic lesions in both lungs had significantly increased in size compared to before. The soft-tissue density mass in the anterior-medial basal segment of the left lower lobe has increased in size to 53 x 35 mm.

**Figure 8:**
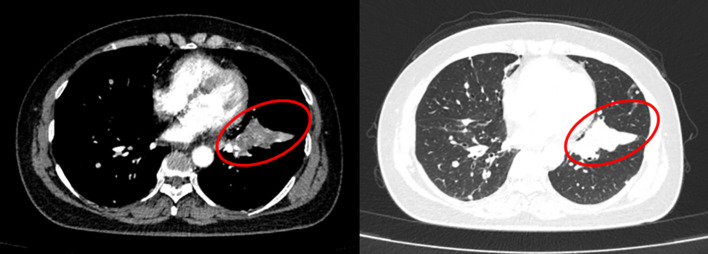
After undergoing two cycles of chemotherapy, the CT scan on July 21, 2025 showed that there was no significant change in the lung lesions compared to the CT scan on June 5, 2025.

In order to stop lung metastases from progressing, the treatment plan was changed to an oral multi-target tyrosine kinase inhibitor (TKI), anlotinib (12 mg once daily, using a 2-week treatment/1-week rest regimen) on August 20, 2025. The patient tolerated anlotinib well. After three cycles of oral anlotinib, the chest CT scan on October 30, 2025, showed that the metastatic lesions in both lungs had decreased in size (**Figure 9**). The response is rated as a partial response (PR). On December 8, 2025, and March 3, 2026, the two chest CT examinations both indicated that the patient remained in the PR state (**Figure 10**). Moreover, the patient’s neurological function remained stable, with normal muscle strength in all limbs and clear speech. She did not report any symptoms such as headache, epileptic seizure, or local sensory disturbance. The brain MRI from February 2025 to March 2026 did not show any signs of intracranial recurrence (**Figure 11**). Currently, the patient remains alive and continues to receive regular and quantified treatment with anlotinib.

**Figure 9:**
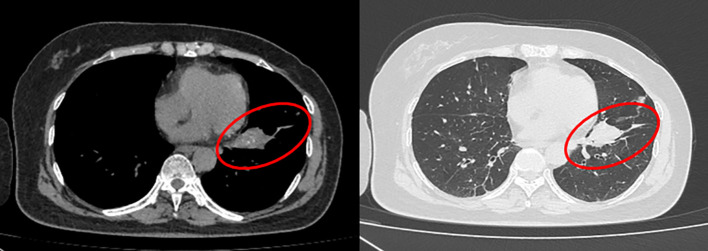
After three cycles of oral anlotinib, a chest CT scan on October 30, 2025, showed a decrease in the size of the bilateral lung metastases.

**Figure 10:**
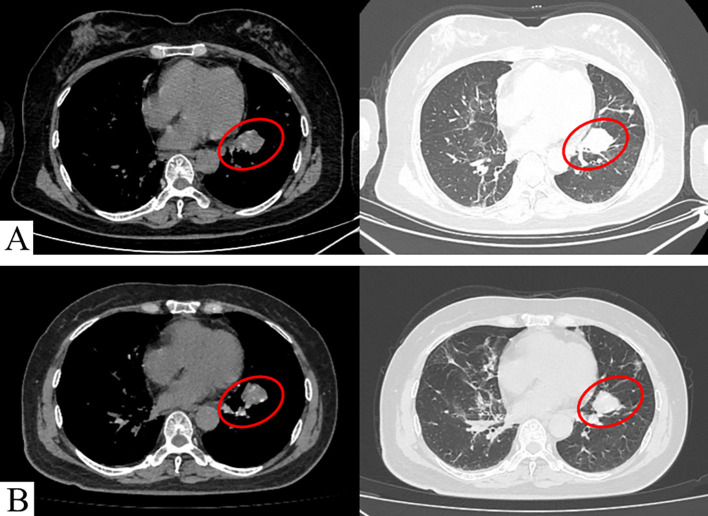
The two chest CT examinations conducted on December 8, 2025 **(A)** and March 3, 2026 **(B)** showed no significant changes in the lung lesions compared to those on October 30, 2025. This suggests that the patient is still in the PR state.

**Figure 11:**
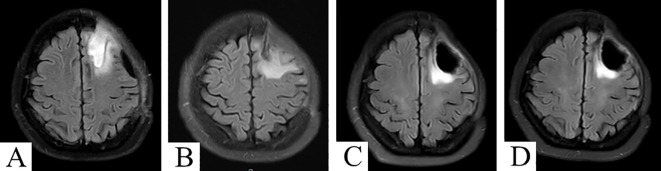
The cranial MRI conducted on February 25, 2025 **(A)**, June 6, 2025 **(B)**, December 10, 2025 **(C)**, and March 3, 2026 **(D)** all indicated no intracranial recurrence.

## Discussion

The pathologic types of malignant thyroid neoplasms are papillary, follicular, medullary and anaplastic. PTC is the most prevalent type, accounting for 65–80% (8). PTC is a neoplasm with a favorable prognosis, as it is well-differentiated and typically exhibits low rates of lymph node infiltration, vascular invasion, or distant metastasis (9). In recent decades, PTC has exhibited a persistent upward trend globally, particularly in developed countries and regions. Although PTC generally manifests as indolent behavior, with a favorable prognosis and a 10-year survival rate exceeding 90%, certain cases demonstrate aggressive features leading to recurrence and metastasis, significantly impacting prognosis and quality of life (10). Within a decade, 70% of PTC patients die from distant metastasis (11). Approximately 11% of PTC cases may develop distant invasion to the lungs and bones, while 1% may involve the brain (12). This article reports an extremely difficult and uncommon clinical case: After a six-year period without disease, a patient who had a total thyroidectomy for PTC developed lung and brain metastases. After conventional treatments failed, the patient ultimately received anlotinib treatment and achieved PR. The uniqueness of this case provides several important clinical insights.

## Treatment decisions for brain lesions

In this case, the lesion in the left frontal lobe was surgically removed. The main reason for this clinical decision was as follows: 1) the patient exhibited obvious neurological dysfunction, requiring immediate decompression treatment; 2) imaging examinations revealed a single and easily resectable lesion; 3) the patient’s physical condition was good (KPS 80), and there were no uncontrollable systemic diseases; 4) surgical resection could provide clear histopathological and immunohistochemical diagnostic results, which was crucial for guiding subsequent systemic treatment.

Moreover, SRS is specifically recommended by the 2025 ATA guidelines as the primary treatment for metastases of the central nervous system (5). Postoperative review of cranial MRI confirmed that the left frontal lobe metastasis had been completely removed, with no remaining enhanced tumor. Additionally, the patient’s weakness in the right limb had significantly improved, her dysarthria had completely disappeared, and she could walk independently on the fifth day after surgery, without any new neurological dysfunction.

There is evidence that postoperative stereotactic radiotherapy of the surgical cavity can decrease local recurrence in patients with a single fully resected brain metastasis, but it does not increase overall survival when compared to simple observation (13). Additionally, the NCCN guidelines state that, it is also acceptable to observe following complete resection of a single brain metastasis in certain patients who have favorable prognostic factors (14). Given that the patient’s physical condition was maintained (KPS 80), the condition was under control at that time, and regular cranial MRI could be monitored. Therefore, after MDT discussion, it was decided to regularly review MRI for monitoring, without performing adjuvant SRS or WBRT.

## Diagnostic reasoning and differential diagnosis

For the diagnosis of thyroid metastatic carcinoma in this patient, it is necessary to systematically rule out other possible causes of isolated brain lesions and multiple pulmonary nodules.

1. For the left frontal lobe mass, the main differential diagnoses include: 1) Metastatic thyroid cancer. Given the patient’s history of PTC, immunohistochemistry after surgery shows that Pax-8, Tg, and TTF-1 are all positive. Although PTC brain metastasis is rare, the presence of bilateral multiple pulmonary nodules in this patient greatly increases the possibility of systemic metastasis (15). 2) Primary brain tumors, including glioblastoma (which typically presents with more prolonged neurological prodromes and infiltrative growth on imaging) and meningioma (which is usually an extra-axial lesion accompanied by a ‘dural tail sign’ not observed in this case (16)). 3) Metastasis of a second primary tumor (lung adenocarcinoma, breast cancer, melanoma). Among them, lung adenocarcinoma is the most common cause of brain metastasis. However, the immunohistochemical results and imaging features do not match, so it was excluded.
2. For multiple pulmonary nodules, the main differential diagnoses include: 1)Primary lung cancer. Usually, it presents as a non-regularly bordered main lesion, whereas this patient has multiple bilateral circular nodules, a typical manifestation of metastatic disease. Secondly, the immunohistochemical results clearly indicate that it originates from the thyroid rather than lung adenocarcinoma (lung adenocarcinoma should be TTF-1 positive and Pax-8 negative) (17). In addition, the biopsy of the pulmonary nodules confirmed that they are metastatic PTC, ruling out the possibility of primary lung cancer. 2) Granulomatous tuberculosis. The pulmonary cancer metastasis to the lungs may present as granular or imaging features in chest X-rays and CT examinations, which are almost indistinguishable from granulomatous tuberculosis (18). Moreover, there have been several reports of cases where thyroid cancer with lung metastasis was initially misdiagnosed as tuberculosis (19, 20). For this patient, there were no corresponding systemic symptoms (fever, night sweats, weight loss), so granulomatous tuberculosis was excluded. 3) Infection. Since there were no signs of infection, such as fever, leukocytosis, or inflammatory markers (such as C-reactive protein), MRI did not show the smooth edge enhancement characteristic of abscesses. The possibility of infection is relatively low.

The diagnostic assessment of this case concludes by highlighting the significance of a thorough multidisciplinary treatment strategy that incorporates pathological diagnosis, imaging characteristics, clinical history, and immunohistochemical testing. The “gold standard” for verifying the source of thyroid follicular cells in metastatic lesions in pathology is the identification of Pax-8, Tg, and TTF-1. This is essential for distinguishing metastatic thyroid cancer from other conditions in differential diagnosis.

## Treatment strategy evolution

¹³¹I is the recommended treatment for patients with PTC and distant metastases, according to the 2025 ATA guidelines (5). However, SPECT/CT imaging after ¹³¹I treatment in this case showed multiple nodules in both lungs, with no significant radioactive iodine uptake and no significant changes on chest CT. According to the guidelines, these results meet the RAIR definition criteria, and patients with RAIR status should not receive additional empirical ¹³¹I treatment and should be prioritized for other treatment options.

The guidelines strongly recommend conducting tissue-based biomarker testing before initiating systemic treatment for progressive diseases to identify actionable oncogenic driver mutations in RAIR-DTC. Targeted therapy and the use of immune checkpoint inhibitors were not possible for this patient because ctDNA analysis showed an ambiguous RET p.G849S variant, no actionable mutations in EGFR, ALK, BRAF, or KRAS, and a pMMR/MSS phenotype. Despite its current classification as a variant of uncertain significance (VUS), the RET p.G849S mutation was detected at a relatively high allele frequency (46.72%) in this patient. This finding raises the possibility that it is a potential clonal driver alteration in the metastatic lineage, highlighting the need for future functional validation studies to elucidate its biological and clinical relevance. Further drug genomics analysis indicated that the platinum, taxane, and pemetrexed regimens showed good predictive efficacy. Although the guidelines acknowledged that the efficacy of traditional cytotoxic chemotherapy for RAIR-DTC patients was poor, the genomic research provided a basis for using the albumin-bound paclitaxel combined with carboplatin for this patient. In this case, the patient’s severe chemotherapy-related toxicity prevented them from continuing after three treatments, necessitating a further modification of the treatment plan.

For patients with progressive RAIR-DTC who lack actionable driver gene mutations, the 2025 ATA guidelines recommend TKIs as the standard first-line systemic treatment. The preferred drug is lenvatinib, and sorafenib is the alternative. Cabozantinib is recommended as the second-line treatment drug. However, in this case, after a thorough MDT discussion, anlotinib was selected. The reasons for this choice are multifaceted, and a comparison summary with the TKIs recommended by the guidelines is as follows (**Table 1**). Firstly, the toxic reactions of anlotinib mainly manifest as hand-foot skin reactions, hypertension, and manageable bone marrow suppression. In a clinical trial, only 10.5% of patients discontinued treatment due to adverse events (21). While using lenvatinib or sorafenib often accompanies significant adverse events, leading to frequent dose reduction and discontinuation. Studies have found that the proportion of patients receiving lenvatinib treatment and experiencing treatment-related adverse reactions was 97.3%, with 75.9% experiencing grade 3 or higher adverse reactions (22, 23). In contrast, anlotinib has better safety, with an intermittent dosing pattern of “take for 2 weeks and stop for 1 week”, facilitating toxicity recovery. Considering that the patient had just completed chemotherapy, anlotinib may have better long-term tolerability in the maintenance stage after chemotherapy and be better matched to the patient’s current physical condition. Secondly, in a clinical trial, anlotinib significantly prolonged the median progression-free survival (mPFS) from 8.4 months in the placebo group to 40.5 months, and was well tolerated. Finally, in the 2021 version of the Chinese Clinical Oncology Society (CSCO) Diagnosis and Treatment Guidelines for Differentiated Thyroid Cancer, anlotinib has been listed as a recommended medication for recurrent/metastatic RAIR-DTC. And the Chinese FDA has approved anlotinib as a new option for treating RAIR-DTC.

**Table 1:** Comparison of multi-target TKIs for RAIR-DTC.

Characteristic	Lenvatinib	Sorafenib	Cabozantinib	Anlotinib
Key experiment	SELECT (Phase III) (22)	DECISION (Phase III)	COSMIC-311 (Phase III)	ALTER01032 (Phase II)
Corresponding guidelines	First-line preferred (ATA 2025)	First-line standard (ATA 2025)	Second-line (ATA 2025)	CSCO guideline-endorsed
Median PFS	18.3 months	10.8 months	Not reached (vs. 1.9 months placebo)	40.5 months
ORR	64.8%	12.2%	15%	59.2%
Key grade ≥3 AEs	Hypertension (42%), proteinuria, diarrhea	Hand-foot skin reaction, diarrhea	Hand-foot skin reaction, diarrhea, fatigue	Hand-foot skin reaction, hypertension, manageable myelosuppression
Dose reduction/interruption	82.4%/67.8%	66.2%/64.3%	56%	~10.5%

The series of treatment decisions for this patient represents a dynamic process of moving from “standard treatment” to “individualized treatment”, fully in line with the core principles of the 2025 ATA guidelines. The sustained PR achieved with this method underscores the value of personalized treatment for complex metastatic PTC.

## Findings from the literature: simultaneous brain and lung metastases in PTC

We also reviewed previously reported cases of PTC with brain and lung metastases. As shown in **Table 2**, among the 14 patients with available data, 3 patients (21%) had both lung and brain lesions at the time of initial diagnosis. The median latency from the initial thyroidectomy to the occurrence of distant metastasis for the remaining 11 patients was 49.6 months (range 12–108 months). The six-year latency of our patients fell fully within the range reported in this study and was consistent with the natural course of classic PTC, further confirming the unpredictability of distant metastasis in PTC. However, it also highlighted the need to include chest and brain imaging in late monitoring for patients with relevant risk factors. Regarding histological subtypes, only one case was definitively classified as high-cell type, further corroborating the notion that classic PTC can also trigger simultaneous lung and brain metastases. With the exception of this outlier, all patients underwent total thyroidectomy. Thirteen patients received high-dose ^131^I ablation therapy, while eight patients received adjuvant treatment with SRS or WBRT. Despite the utilization of diverse treatment modalities, survival data were available for a mere nine patients: Six patients survived (follow-up ranging from five to 204 months), two patients died within one year of brain metastasis diagnosis, and one patient died 17 years later. This finding suggests that, while long-term survival is possible (particularly when stereotactic radiosurgery is feasible), rapid neurological progression remains a potential outcome.

**Table 2:** Synchronous lung and brain metastases from PTC reported previously in the literature.

Case	Author	Year	Age	Gender	Site of metastasis	Treatment	TNM classification after operation	Survival after diagnosis of metastasis	Family history of thyroid disease	Exposure to external or accidental radiation
1 (24)	Knight et al.	2022	73	Female	Lungs, Brain	Surgical+Radiotherapy+RAI ablation(131I)	NA	17 years	NA	NA
2 (25)	Tunio et al.	2015	72	Female	Lungs, Brain, Bones	Surgical+Radiotherapy+RAI ablation(131I)	pT2N1aM0	6 months	NA	NA
3 (25)	Tunio et al.	2015	64	Male	Lungs, Brain	Surgical+Radiotherapy+RAI ablation(131I)	pT3N0M0	Alive	NA	NA
4 (26)	Guelho et al.	2016	43	Female	Lungs, Brain	Surgical+Radiotherapy+RAI ablation(131I)	pT4N1bMx	Alive	NA	NA
5 (27)	Ha et al.	2023	66	Female	Lungs, Brain, Bones	Stereotactic surgery (SRS) +RAI ablation(131I)	pT1N1M1	Alive	NA	NA
6 (27)	Ha et al.	2023	40	Female	Lungs, Brain, Bones	Surgical+Radiotherapy+RAI ablation(131I)+Stereotactic surgery (SRS)	pT4N1b	Alive	NA	NA
7 (27)	Ha et al.	2023	33	Female	Lungs, Brain	Surgical+Radiotherapy+RAI ablation(131I)	cT4aN1bM1	Alive	NA	NA
8 (28)	Bruglia et al.	2009	44	Male	Lungs, Brain, Mediastinum, Muscle	Surgical+Radiotherapy+RAI ablation(131I)+Chemotherapy	NA	1 years	NA	NA
9 (29)	Ramos et al.	2021	64	Female	Lungs, Brain	Stereotactic surgery (SRS)	NA	Alive	NA	NA
10 (30)	Krajewska et al.	2010	55	Male	Lungs, Brain, Muscles, Liver	Surgical+Radiotherapy+RAI ablation(131I)+Chemotherapy	pT3aNxM0	17 years	NA	NA
11 (31)	Cho et al.	2014	53	Female	Lungs, Brain, Mediastinum, Bones	Surgical+Radiotherapy+RAI ablation(131I)	NA	NA	NA	NA
12 (32)	Xu et al.	2011	46	Female	Lungs, Brain	Surgical+Radiotherapy+RAI ablation(131I)+Stereotactic surgery (SRS)	NA	Alive	NA	NA
13 (33)	Cross et al.	2008	29	Female	Lungs, Brain	Surgical+RAI ablation (131I)	NA	Death	NA	NA
14 (34)	Dobrorodniy et al.	2020	59	Female	Lungs, Brain, Bones	Surgical+Stereotactic surgery (SRS) +RAI ablation(131I)	NA	Alive	NA	NA

According to the literature, middle-aged women are more likely to have lung and brain metastases at the same time in PTC. The patient characteristics and treatment patterns described in the literature align with this case. This implies that, even in cases where thyroid cancer has spread to multiple organs, we should use a combination of systemic and local therapy to achieve significant disease control.

## Limitations

Although this case offers insightful information, it has certain drawbacks. Firstly, the results have limited generalizability because this is a retrospective single-case study. Large-scale prospective clinical trials are still needed to confirm the efficacy and safety of the sequential treatment approach (chemotherapy followed by anlotinib) outlined in the report. Secondly, due to the retrospective nature of this study, the data integrity is limited in some respects. The patient received treatment at multiple medical institutions, which prevented the acquisition of complete longitudinal data on serum Tg and TgAb levels. The quantitative characterization of the shift from remission to distant metastasis is limited by the lack of serial Tg/TgAb trends, which are crucial biomarkers for tracking recurrence in differentiated thyroid cancer. Thirdly, the clinical significance of the detected RET p.G849S variant remains classified as a VUS. Although its high abundance (46.72%) suggests a clonal event, functional validation or paired analysis with the primary tumor tissue was not performed, making it impossible to determine whether this variant represents a true driver mutation or a passenger event that emerged during metastasis. Lastly, even though the patient has achieved a long-lasting partial remission, the follow-up time is still relatively short. In order to evaluate the long-term viability of this therapeutic approach, longer-term observation is required to evaluate long-term progression-free survival, overall survival, and the potential emergence of acquired resistance to anlotinib.

## Conclusions

In conclusion, we reported a case of brain and lung metastasis discovered 6 years after thyroid cancer surgery. Several important clinical points about the treatment of PTC were further supported by this case. First, PTC can present with advanced multi-organ metastasis even in cases with a long disease-free interval; this emphasizes the significance of performing early chest and brain imaging examinations. Second, this case demonstrated the clinical value of molecular analysis based on ctDNA in RAIR-DTC, which not only helps rule out ineffective targeted or immunotherapy regimens but also enables the determination of feasible chemotherapy strategies through pharmacogenomic analysis, thereby guiding truly personalized treatment. This case also highlights the need to constantly look for new targets in the field of precision oncology for thyroid cancer because some RAIR-DTC patients do not have operable driver mutations. Lastly, this case provided a real-world example of the 2025 ATA guidelines’ principle: TKIs should be started for progressive RAIR-DTC cases without operable mutations. New clinical evidence, the patient’s unique toxic history, and local guidelines should all be taken into consideration when modifying the treatment plan. Anlotinib was substituted for chemotherapy when treatment tolerance was low, leading to long-lasting PR.

## Data Availability

The original contributions presented in the study are included in the article/supplementary material. Further inquiries can be directed to the corresponding author.
